# Phenotypic Heterogeneity of Triple-Negative Breast Cancer Mediated by Epithelial–Mesenchymal Plasticity

**DOI:** 10.3390/cancers13092188

**Published:** 2021-05-02

**Authors:** Barbora Kvokačková, Ján Remšík, Mohit Kumar Jolly, Karel Souček

**Affiliations:** 1 Department of Cytokinetics, Institute of Biophysics of the Czech Academy of Sciences, 612 65 Brno, Czech Republic; kvokackova@ibp.cz; 2 International Clinical Research Center, St. Anne’s University Hospital, 656 91 Brno, Czech Republic; 3 Department of Experimental Biology, Faculty of Science, Masaryk University, 625 00 Brno, Czech Republic; 4 Human Oncology & Pathogenesis Program, Memorial Sloan Kettering Cancer Center, New York, NY 10065, USA; remsikj@mskcc.org; 5 Centre for BioSystems Science and Engineering, Indian Institute of Science, Bangalore 560012, India; mkjolly@iisc.ac.in

**Keywords:** triple-negative breast cancer, plasticity, epithelial–mesenchymal transition, mesenchymal–epithelial transition, metastasis

## Abstract

**Simple Summary:**

Epithelial–mesenchymal transition (EMT) and its reverse process mesenchymal–epithelial transition (MET) are considered critical events in the cancer progression. These programs are tightly connected with the development of metastasis–the lethal stage of the disease. Both EMT and MET shape the biology of unusually aggressive and heterogeneous triple-negative breast cancer (TNBC). In this review, we summarize the current knowledge of EMT/MET plasticity in the context of TNBC, with a special focus on drivers and mechanisms behind these processes.

**Abstract:**

Triple-negative breast cancer (TNBC) is a subtype of breast carcinoma known for its unusually aggressive behavior and poor clinical outcome. Besides the lack of molecular targets for therapy and profound intratumoral heterogeneity, the relatively quick overt metastatic spread remains a major obstacle in effective clinical management. The metastatic colonization of distant sites by primary tumor cells is affected by the microenvironment, epigenetic state of particular subclones, and numerous other factors. One of the most prominent processes contributing to the intratumoral heterogeneity is an epithelial–mesenchymal transition (EMT), an evolutionarily conserved developmental program frequently hijacked by tumor cells, strengthening their motile and invasive features. In response to various intrinsic and extrinsic stimuli, malignant cells can revert the EMT state through the mesenchymal–epithelial transition (MET), a process that is believed to be critical for the establishment of macrometastasis at secondary sites. Notably, cancer cells rarely undergo complete EMT and rather exist in a continuum of E/M intermediate states, preserving high levels of plasticity, as demonstrated in primary tumors and, ultimately, in circulating tumor cells, representing a simplified element of the metastatic cascade. In this review, we focus on cellular drivers underlying EMT/MET phenotypic plasticity and its detrimental consequences in the context of TNBC cancer.

## 1. Introduction

Triple-negative breast cancer (TNBC), accounting for approximately 10–15% of all breast carcinomas, associates with an earlier age of onset and frequently manifests inherently aggressive clinical behavior [1,2]. TNBC is characterized by profound heterogeneity and absence of the expression of classical breast cancer markers: estrogen (ER), progesterone (PR), and human epidermal growth factor 2 (HER2) receptors. For that reason, TNBC patients do not benefit from therapies targeting estrogens, progestins and HER2, and conventional chemotherapy and radiotherapy often remain the only treatment option [3,4,5,6]. Based on PAM50 intrinsic subtype classification (Basal-like, HER2-enriched, Luminal A, Luminal B, Normal-like, Claudin-low), most of clinical TNBC samples harbor basal like-signature [7,8]. Further comprehensive molecular studies dissected TNBC diversity and characterized 4 distinct subtypes, each displaying unique disease biology: basal-like 1, basal-like 2, mesenchymal, and luminal androgen receptor type [9,10]. Besides cancer cells, the TNBC tumor mass composes of extracellular matrix and different stromal populations, such as immune and endothelial cells, fibroblasts and others, collectively known as the tumor microenvironment (TME) [11]. TME plays a crucial role in all stages of carcinogenesis, including tumor growth, immune evasion, therapy resistance, and metastatic dissemination [12,13,14,15]. Intratumoral heterogeneity is determined by the phenotypic and molecular diversity within the tumor and can be explained by several concepts, including the differentiation state of the cancer cell of origin, mechanisms of cell plasticity, dynamic genomic alterations, and clonal evolution and selection [16,17,18]. One of the key mechanisms that contributes to phenotypic plasticity and heterogeneity is the epithelial–mesenchymal transition (EMT) and its reverse program known as mesenchymal–epithelial transition (MET). Both of these fundamental programs play specific roles in embryonic development and adult tissue homeostasis. During EMT, cells can undergo dynamic transitions from an epithelial (E) state, characterized by the presence of cell-to-cell junctions, apical-basal polarity, and interactions with basement membrane to a mesenchymal (M) phenotype, identified by fibroblast-like morphology linked with increased migratory and invasive properties [19,20]. Aberrant activation of EMT by cancer cells that enhances their invasive and motile behavior represents a critical event in tumor progression. In this context, EMT was shown to facilitate the spread of cancer cells from primary tumor to circulation, followed by extravasation and dissemination to secondary sites [21,22]. On the other hand, reversion of cells back to epithelial state during MET enables tumor cells to colonize distant organs with higher efficiency and form macrometastasis [23,24]. Apart from their roles in metastatic progression, both of these programs influence therapy resistance, prevent senescence, enhance survival and foster entrance of both normal and neoplastic mammary epithelial cells into stem-cell states [25,26,27,28]. Malignant cells have the capacity to dynamically switch between EMT and MET, which allows them to exist in a wide array of hybrid E/M states, co-expressing both epithelial and mesenchymal markers and partially bearing features of both phenotypes. Some of these highly plastic and aggressive hybrid phenotypic states provide cancer cells with increased fitness and flexibility required for tumor development. These states are believed to be generated in a stepwise manner through the E/M continuum and strongly depend on specific biological context [29,30,31,32,33,34,35]. According to a recent consensus statement, the favored term for the ability of cancer cells to adopt mixed E/M features is epithelial–mesenchymal plasticity (EMP) [20]. While the role and importance of EMT in tumor progression have been widely studied and emphasized in the past, hybrid phenotypes and MET did not get such attention.

According to recent classification, the most commonly used TNBC cell lines in discussed studies possess basal (MDA-MB-468, HCC1806) or mesenchymal phenotype (MDA-MB-231, BT-549, SUM-159 and HS578T) [36]. In addition, few studies employed also non-transformed MCF10A and HMLE cell lines that are immortalized and non-tumorigenic cells per se; however, they can exhibit features similar to TNBC cancer cell lines, including the lack of hormone receptors and HER2 expression [37,38].

### Insights into TNBC Heterogeneity

Apart from lack of reliable biomarkers, TNBC tumors exhibit a heterogeneous clinical behavior associated with poor prognosis; therefore, a concerted effort has been undertaken to decipher TNBC heterogeneity. On a single-cell level, five distinct subgroups of malignant cells shared by tumors have been identified, including one clinically relevant subpopulation characterized by multiple signatures of treatment resistance, metastasis, activation of glycosphingolipid metabolism, and associated innate immunity sensing and inflammation [39]. Similarly, in matched primary tumors and micrometastases from basal-like TNBC patient-derived xenografts (PDX) models, a profound transcriptional diversity among tumor cells was observed, as individual tumor clusters associated with genes related to fatty-acid metabolism, proteasome function, extracellular matrix (ECM) modulatory signature, or EMT gene signature [40]. Moreover, scRNA-seq revealed transcriptional reprogramming of tumor cells undergoing chemotherapy pressure of docetaxel and epirubicin [41]. On the protein level, imaging mass cytometry identified tumor populations with high levels of Ki-67, p53, EGFR, and CAIX; basal cytokeratins; and luminal cytokeratins [42]. Besides tumor cells with a basal phenotype characterized by SMA expression, Vimentin, and EMT-like tumor cells, TNBC clinical specimens also consist of fibroblasts, endothelial and immune cells [43]. Immune cells, particularly tumor-infiltrating lymphocytes are often implicated in TNBC prognosis [44,45] and such a complex immune landscape is composed of specific B cell populations, tissue macrophages and T-lymphocytes with distinct transcriptomic signatures including naïve/early costimulatory, exhausted, or cytotoxic [46]. In the context of stromal cells, usually represented in tumors by cancer-associated fibroblasts (CAFs) [47,48], Wu and colleagues identified distinct subsets of myofibroblast-like and inflammatory CAFs along with differentiated and immature perivascular-like stromal cells [15]. Myofibroblastic and inflammatory fibroblasts comprised bulk tumors in the different patient cohort although sc-RNAseq analysis was performed on already presorted CAF subset defined by FAP^hi^ CD29^med-hi^ SMA^hi^ expression [49].

In addition to extensive intratumoral heterogeneity, TNBC patients display a heterogeneity in somatic mutations and copy number aberrations (CNA) [8] and this mutational profile is tightly connected with TNBC subtype [18,50]. For example, TP53 is more frequently mutated in basal-like TNBC, with enriched nonsense and frameshift mutations and along with PIK3CA and PTEN is involved in the early stages of breast cancer development [18,50]. In addition, metastatic TNBC demonstrate enrichment of driver and targetable CNA relative to primary tumors [51] and majority of patients exhibit complex aneuploid rearrangements [52,53,54].

Nevertheless, these studies provide mostly descriptive data of TNBC cellular and genomic heterogeneity, and further mechanistic studies are needed to functionally link particular populations with EMP.

## 2. Mechanisms Regulating Epithelial–Mesenchymal Plasticity

**Figure 1:**
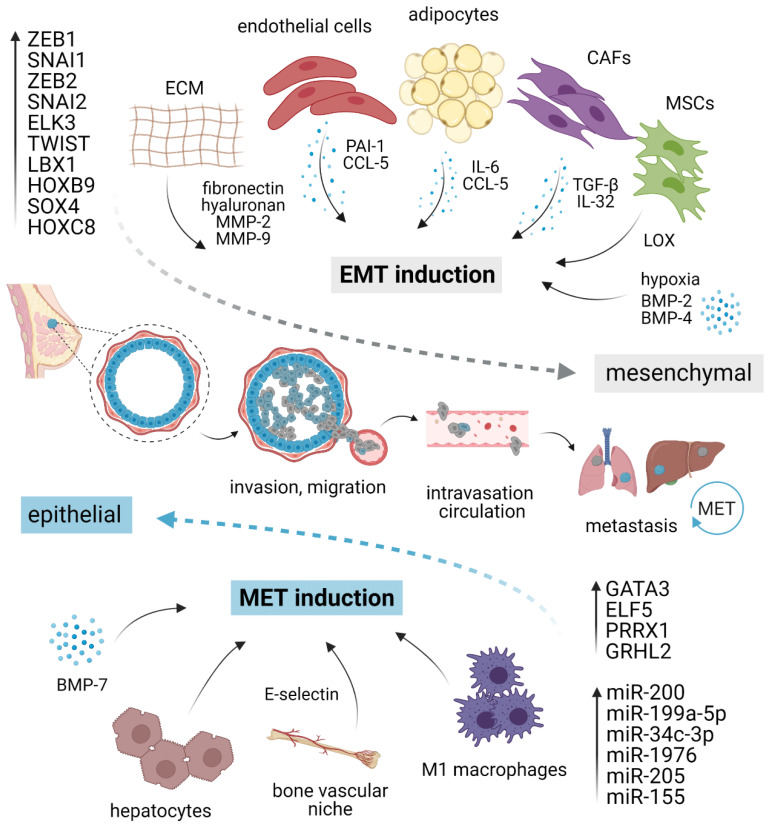
EMT/MET plasticity in breast cancer. EMT is induced by pro-inflammatory cytokines and molecules secreted by different stromal cells present in the tumor microenvironment, ECM elements, and hypoxia. Different cell types such as macrophages or hepatocytes might activate MET. Each program is executed via a specific set of transcription factors and/or corresponding miRNAs. The activation of EMT and mesenchymal phenotype (gray) grants cancer cells the ability to migrate, invade, intravasate, survive in circulation, and extravasate at distant sites. At the secondary organs, mesenchymal cells revert to an epithelial state (blue) through MET, regaining the ability to form macrometastasis. EMT, epithelial–mesenchymal transition; MET, mesenchymal–epithelial transition; CAFs, cancer-associated fibroblasts; MSCs, mesenchymal stromal cells; ECM, extracellular matrix; MMP, matrix metalloproteinase; LOX, lysyl-oxidase. Created with BioRender.

Owing to the transient and dynamic nature of EMT/MET during invasion, dissemination, and metastatic outgrowth, a growing body of evidence suggests that EMP in cancer cells is fueled by the external microenvironmental cues from primary TME or pre-existing metastatic niche, rather than driven by the cell-intrinsic genetic alterations [23]. A complex network of stimuli converges inside the nucleus to up- or down-regulate genes required for epithelial or mesenchymal characteristics, including those involved in migration, invasion, and stemness (Figure 1).

### 2.1. Microenvironmental Stimuli

Multiple studies uncovered many microenvironmental factors in promoting EMT/MET machinery, including proinflammatory cytokines secreted by stromal cells, local hypoxic gradients, or specific components of ECM [55]. The vast majority of well-validated EMT inducers are pleiotropic growth factors or cytokines, including EGFs, FGFs, and numerous interleukins, such as IL-6 and IL-8 [55,56]. The most potent EMT inducer is TGF-β (Transforming growth factor-beta), which activates members of the SMAD family of signal transducers. TGF-β can also activate other pathways that are collectively referred to as “non-canonical” TGF-β signaling that complements SMAD action [57,58]. Besides activation of EMT program in epithelial cells, TGF-β promotes expression of cancer stem cells (CSCs) drivers and markers, and together with canonical and non-canonical WNT ligands function in an autocrine manner to maintain mesenchymal state [27,59]. TGF-β can further induce cell invasion through upregulation of Matrix metalloproteinase (MMP)-2 and -9 [60]. In a paracrine manner, TGF-β sculptures local TME and supports immunosuppression [61,62,63,64,65]. Plasma-derived TGF-β-related protein signature consisting of CLIC1, MAPRE1, SERPINA3 genes was predictive of TNBC tumor progression [66]. On the contrary, BMP-7 (Bone morphogenetic protein-7), a TGF-β family member, often acts as a TFG-β antagonist and preserves epithelial phenotype by counteracting SMAD2/3-dependent TGF-β signaling through BMP SMADs 1/5/8. Buijs et al. demonstrated that BMP-7 overexpression in cancer cells or systemic BMP-7 introduction in vivo resulted in enhanced epithelial phenotype and inhibition of bone metastasis development. However, exogenous BMP-7 exposure alone was not sufficient to restore E-cadherin expression. Therefore, it remains unclear whether the decreased growth of bone metastasis is orchestrated by the lack of E-cadherin, as discussed later, or depended on specific bone microenvironment regardless of E-cadherin status [67]. Mechanistically, BMP-7 suppresses cancer cell invasion by inhibiting TGF-β-induced Integrin β3 [68]. These findings were independently confirmed by a different group that further observed EMT attenuation after treatment with nanoparticles targeting Integrin β3 [69]. On the other hand, BMP-2 and BMP-4 were reported as EMT inducers, acting via Retinoblastoma/CD44 and Notch signaling, respectively [70,71].

Although several studies suggested a potential function of resident adipocytes in TNBC plasticity, their role in EMT/MET has not been satisfactorily elucidated yet. For instance, tumor cells exposed to mature adipocytes displayed accelerated invasive properties and enhanced lung metastasis potential [72]. In the experimental settings, human adipose stem cells (ASC) stimulated breast cancer motility and invasion through production of CCL5, as a response to cancer cells [73,74]. Strikingly, CCL5 is detectable in peritumoral adipose tissue of TNBC patients and correlates with lymph node and distant metastases, and poor patient outcome [73]. Sabol and colleagues highlighted the role of obesity in cancer progression [75,76]. Compared to lean donors (BMI < 25), ASC from obese donors (BMI > 30) activated EMT resulting in elevated number of circulating tumor cells (CTC) and increase in metastasis incidence in PDX via leptin signaling but surprisingly did not influence the tumorigenesis of TNBC xenografts [76]. Moreover, adipocyte-derived IL-6 induced EMT through STAT3 signaling [77]. Other stromal cell types capable of inducing EMT include cancer-associated fibroblasts employing paracrine TGF-β [78,79] or IL-32 and Integrin β3–p38 MAPK signaling [80], mesenchymal stromal cells producing lysyl oxidase [81], and endothelial cells via PAI-1 (SERPINE1) and CCL5 signaling [82].

Regarding ECM elements, exogenous fibronectin can accelerate EMT through the activation of Src kinase and ERK/MAP kinase signaling [83]. Alternatively, elevated production of ECM component hyaluronan was sufficient to induce EMT, employing the PI3K/Akt cell survival pathway [84]. Lastly, EMT can occur as a response to a hypoxic environment [85,86], mechanistically observed as a result of hypoxia-induced activation of Notch ligand Jagged2 [87] or governed by Carbonic anhydrase IX [88,89].

The first evidence for direct MET induction associated with E-cadherin restoration in response to microenvironmental cues was provided by Chao et al. In their experimental model, hepatocytes induced the E-cadherin re-expression in otherwise mesenchymal MDA-MB-231 cell line through passive loss of E-cadherin promoter methylation [90,91]. Such E-cadherin re-expression and concomitant quiescent epithelial phenotype also arose after co-cultivation of cancer cells with M1 macrophages [92]. In the bone milieu context, E-selectin from bone vascular niche has been identified as a driver of MET and Wnt activation in cancer cells, promoting bone metastasis [93].

### 2.2. Tumor Infiltrating Lymphocytes

Various cellular and humoral components of the immune system can constrain or promote the growth of tumors through a dynamic process known as immunoediting. Through various mechanisms of immune-mediated plasticity, including EMT [94], cancer cells change their phenotype to edit their immunogenicity and acquire immunosuppressive traits. The contribution of particular immune cell types on cancer cell plasticity is becoming the focus of mechanistic studies especially due to the rise of immunotherapies. The number of tumor infiltrating lymphocytes (TILs) have a prognostic value in TNBC patients that did not receive adjuvant therapy [95]. Increased incidence of TILs in TNBC can be a consequence of increased mutational burden and neoepitope exposure [96]. In particular, cytotoxic CD8 lymphocytes appear to have an antitumor activity [97]. However, this phenomenon can be suppressed by the CD24-/CD44+ /ALDH+ cancer stem cell population [98]. Conversely, Seo and colleagues showed that TNBC infiltration with CD4+ and CD8+ T cells was positively correlated with the presence of CD44^high^CD24^low^ tumor cells and Vimentin expression [99]. The cancer stem cell population that underwent beta-catenin-STT3-mediated EMT manifested an accumulation of immunosuppressive molecule PDL1 [100], a target of several immunotherapies. Independent study further showed that mesenchymal breast cancer cells express decreased levels of MHC I and high levels of PDL1, as well as increased number of tumor-associated macrophages in the tumor stroma.

### 2.3. Transcription Factors

Besides epigenetic stabilization, EMP is largely mediated and maintained by a core set of transcription factors (TFs), most notably SNAI1 (known as Snail), SNAI2 (known as Slug), Twist-related protein 1 (TWIST1), and Zinc-finger E-box-binding homeobox-1 and -2 (ZEB1 and ZEB2, respectively) [19,101]. Expression of these EMT-TFs is often selective, context- and cell type-specific, and transient, and their deregulation in different E/M states imply a high degree of malignant cell plasticity [102]. ZEB1 represents a central switch that induces and determines EMT and is considered and studied as a potential biomarker for poor clinical outcome in triple-negative breast cancer [55,103,104,105,106]. Besides the induction of EMT, activation of ZEB1 by TGF-β can fuel de novo generation of CSCs from non-CSCs populations with poised chromatin within the ZEB1 promoter region and is critical for the maintenance of cells in CSCs-like, CD44^hi^ state [103]. On the other hand, ZEB1 downregulation mediated by PKCα leads to inhibition of mesenchymal phenotype, cell migration, and invasiveness [107]. ZEB1 is known to cooperate with ELK3, a ternary complex factor from the ETS TFs family, to repress E-cadherin by transcriptional activation of ELK3 expression [108]. Suppression of ELK3 through epigenetic activation of GATA3 rendered cancer cells unresponsive to TGF-β and led to their reprogramming into a non-invasive, cuboidal-like, epithelial state, as observed both in vitro and in vivo [109]. In concordance with these findings, a similar effect on epithelial phenotype has been reported for GATA3, a TF considered a master suppressor of metastasis and associated with better prognosis [110]. GATA3 restored E-cadherin expression by binding into GATA-like motifs located within the E-cadherin promoter. Its overexpression reduced the levels of mesenchymal markers Vimentin, N-cadherin, and MMP-9, and resulted in the formation of smaller tumors without overt metastasis [111,112]. Another well-described MET inducer from the ETS family, ELF5, can reactivate epithelial state through transcriptional repression of SNAIL2, a prominent EMT inducer [113]. However, to repress the epithelial branch of EMT program, SNAI2 requires concurrent induction and cooperation with TWIST1 [114]. Similarly, knockdown of SNAI2 alone failed to revert EMT and suppressed the tumor initiation, in contrast to more potent effects associated with loss of SNAI1 [28]. This observation suggests that in TNBC, SNAI2 requires cooperation with other molecules and is insufficient to modulate EMP alone. In other words, SNAI2/Slug seems to induce a weaker EMT than SNAI1/Snail [115,116].

There is growing evidence implying the role of several members of the homeobox (HOX) TF family [117,118,119]. HOXB9 has been reported to promote the mesenchymal state, tumor growth, angiogenesis, and distant metastasis to the lung [120]. Similarly, HOXC8 functions as an EMT and migration inducer through transcriptional activation of MGP (Matrix Gla protein) [121]. Ladybird homeobox 1 (LBX1) displayed an analogous pattern and accelerated EMT through direct upregulation of ZEB1, ZEB2, SNAI1, and TGF-β2. However, MCF10A cells expressing LBX1 alone were not capable of forming tumors. Only cells co-expressing LBX1 and HRAS^V12^ gave rise to tumors, suggesting oncogenic cooperation with LBX1 [122]. On the contrary, HOX member PRRX1 has the capacity to completely reactivate the epithelial state, even in the presence of TWIST1, and the loss of PRRX1 granted cancer cells the ability to colonize lungs [24]. Another widely reported TF involved in EMT/MET is Grainyhead-like 2 (GRHL2), which plays a fundamental role in the maintenance of epithelial phenotype and wound healing. GRHL2 is capable of suppressing TGF-β- and TWIST1-induced or spontaneous EMT occurring in a subpopulation of CD44^high^CD24^low^ cells within the heterogeneous HMLE cell line [123]. GRHL2 is directly suppressed by ZEB1, which, in turn, represents a direct target for GRHL2-driven repression, suggesting that GRHL2 and ZEB1 form a double-negative regulatory feedback loop [124]. Moreover, GRHL2 expression is lost at the invasive front of primary human breast tumors and cancer cells disseminated into lymph nodes [125].

The extent of EMT and minimal necessary duration of EMT-TF expression is likely to be TF-dependent, possibly because the dynamic trajectories of EMT in the multi-dimensional landscape may or may not overlap [126]. A specific set of TFs appears to be important for rapid induction of TGF-β-mediated EMT but fails to fully complete and maintain classical EMT. This was demonstrated in a study concerning developmental factor SOX4 [127]. Intriguingly, even the transient TWIST1 expression was sufficient to induce stemness, promote tumor-initiating features, and elicit stably altered, plastic epithelial cell state [128], consistent with observations that a full-blown EMT may be detrimental for stemness and/or metastasis [129,130,131]. Considering the limited number of oncogenic transcription factors deregulated in human cancer, TFs may serve as promising targets in the future. Although TFs have been historically viewed as undruggable, there are currently several TF inhibitors undergoing preclinical and clinical testing for a various cancer types [132].

### 2.4. MicroRNAs

Recently published data have shown that EMT/MET inducing transcriptional factors often reciprocally interact with microRNAs. MicroRNAs (miRNA) are small, endogenous, non-coding RNAs that regulate gene expression. Their levels are significantly deregulated in human cancers where they might function as either oncogenes or tumor suppressors [133,134]. The miR-200 family is considered to be a “guardian of the epithelial phenotype”, suppressing EMT by directly targeting ZEB1, ZEB2, and other transcriptional repressors of E-cadherin for decay [135,136,137,138]. Reciprocal ZEB/miR-200 feedback loop contributes to EMP and controls central cellular processes [104,139]. Mathematical modeling of this feedback loop can give rise to many interconverting phenotypes—epithelial, mesenchymal, and hybrid E/M [140].

Experimental restoration of miR-200c expression in TNBC cell lines MDA-MB-231, BT549, and SUM159, all of which express miR-200c at relatively low endogenous levels, reversed the EMT gene expression signature, as demonstrated by an increased E-cadherin, decreased ZEB1 and repressed immunosuppressive gene pattern expression [141]. Besides translational repression of ZEB1, miR-200-mediated transcriptional upregulation of E-cadherin was associated with increased acetylation of histone H3 at the E-cadherin promoter [142]. Moreover, the study by Wang et al. demonstrated that an accelerated MET program was orchestrated by an upregulation of miR-200c as a response to tamoxifen exposure that resulted in demethylation of miR-200c promoter [143]. Mechanistically, miR-200 re-expression not only drives epithelial differentiation but also promotes the growth of macrometastasis by directly targeting SEC23A, a regulator of metastasis-suppressive protein secretion, involving Insulin-like growth factor-binding protein 4 (IGFBP4) and Tubulointerstitial nephritis antigen-like 1 (TINAGL1) [144]. Similar results were obtained using isogenic mouse breast cancer models 67NR, 168FARN, 4TO7, and 4T1, that differ in their ability to metastasize when implanted into the mammary fat pad. Only epithelial cell line 4T1, expressing miR-200c and E-cadherin, formed macroscopic metastasis. Mesenchymal cell line 4T07 is capable to disseminate, but forms only micrometastases. Overexpression of miR-200 in 4T07 cell line strikingly augmented MET and facilitated macrometastatic outgrowth [145]. These results collectively point to the possibility that MET induced by the miR-200 family may be crucial for the colonization step, hence necessary for successful completion of the metastatic cascade.

Apart from the miR-200 family, miR-199a-5p overexpression promoted epithelial state, as demonstrated by the reduced cell migration and invasion, as well as increased CDH1 (encoding E-cadherin) and decreased ZEB1 and Twist expression [146]. Additionally, several different miRNAs were identified and described as the regulators of cell plasticity. Increased level of miR-34c-3p resulted in the stimulation of tumor-suppressing features and reversed the EMT through negative modulation MAP3K2 pathway [147]. Similarly, miR-1976 was determined as epithelial “keeper” due to the acceleration of EMT via PIK3CG after its knock-down [148]. miR-205 was, along with miR-200, shown to be significantly reduced in cells undergoing EMT [135]. Finally, miR-155 expression levels inversely correlated with the expression of several EMT markers including SMA, osteonectin, and CD146 in a cohort of TNBC patients. Moreover, miR-155 expression was reported as an independent favorable prognostic factor of distant metastasis-free survival [149].

## 3. Plasticity in TNBC Progression

Although EMT is widely accepted as a key transcriptional program required for tumor invasion and dissemination, the colonization and outgrowth of recruited cells in the distant organs is believed to require an opposite process, known as MET [23,150]. The MET has been supported mainly by histopathological analyses that revealed morphological resemblance and EMP markers expression between primary tumors and matched metastatic lesions, particularly in E-cadherin expression pattern, which was similar or very often increased in lymph node metastases, relative to the matched primary tumors [151,152,153]. Despite these correlative clinical findings, rigorous functional studies linking MET with metastatic colonization ability are scarce. Major obstacles in investigating MET are associated with the lack of dynamic in vivo models and the sporadic nature of invasion and colonization, making these observations statistically improbable to identify. An important notion is that EMT and MET are not binary processes, and MET does not simply mirror EMT; hence the loss of epithelial features in the cell does not necessarily result in rigid, irreversible mesenchymal phenotype. Cancer cells may not revert to being epithelial if they have crossed a ”tipping point” in terms of induction of EMT [154], and can often reside in a hybrid or partial EMT phenotype endowed with combined epithelial (e.g., cell-to-cell adhesion) and mesenchymal (e.g., motility) features [29,150]. Individual cells with partial EMT phenotype do not fully complete the EMT program and co-express both epithelial markers (such as EpCAM, keratins or E-cadherin) and mesenchymal markers (e.g., Vimentin) along with a differentially expressed set of EMT TFs. Such hybrid cells are presumed to have the highest tumorigenicity and metastatic potential [28,32,33,131,155] and were also observed in breast carcinomas, especially in CTCs exhibiting a spectrum of EMP states [155,156,157,158].

### 3.1. Circulating Tumor Cells

Recent technological advances allowed isolation and characterization of CTCs, the precursors of metastasis, and their presence in the blood of cancer patients is closely associated with poor prognosis in many tumor types, including TNBC [159,160,161,162]. Individual CTCs or CTC clusters in blood provide a simplified view to metastatic cascade, particularly during the spread to distant sites [163]. Although many CTCs display mesenchymal phenotype, several studies reported that aggressive CTCs might reside in the hybrid E/M phenotype. One of the first studies identified a fraction of rare bi-phenotypic E/M cells in TNBC clinical specimens based on expression of epithelial markers Keratins 5, 7, 8, 18, and 19, EpCAM, CDH1 and mesenchymal markers FN1, CDH2 and SERPINE1/PAI1 [157]. Consistently with TNBC itself being enriched for mesenchymal-like phenotype, CTCs isolated from blood resided predominantly in M, and to a lesser extent also in M/E states. Additionally, in patients with progressive metastatic disease, the proportion of CTCs that shifts to M/E and M states reflects the response to chemotherapy regimen and disease progression, in contrast to proportional E, E/M, E = M, M/E, M states present at the beginning of treatment [157]. Employing a syngeneic 4T1 mouse model, Liu and colleagues associated EMT phenotypes of CTCs and disseminated tumor cells (DTCs) with their ability to form lung metastasis. CTCs bearing hybrid phenotype, characterized predominantly as epithelial (positive for EpCAM, cytokeratin and E-cadherin) with limited mesenchymal traits defined by Vimentin expression represented the most aggressive cells with the strongest lung metastatic capacity when compared to mesenchymal CTCs [155]. In accordance, higher proportions of EpCAM^+^ cells in the bloodstream of human patients correlated with distant metastases and poor outcome [155]. Similarly, using Vimentin as a mesenchymal marker and cytokeratin as an epithelial marker, CTCs isolated from TNBC PDX models were characterized by mixed E, M, and E/M phenotypes. A unique PDX model rapidly metastasizing to the liver and lungs showed that the great majority of CTCs reflected mixed E/M state [164]. However, a further investigation involving multiple PDX models and human patient cohorts is required to confirm these experimental observations.

### 3.2. MET and Metastasis

The majority of distant metastases in epithelial carcinomas are well-differentiated, resembling primary tumors. Accumulating data indicate that this re-differentiation is fueled by transient rounds of EMT–MET, which endow cells with properties required to successfully complete the metastatic cascade [23]. In presented experimental studies, MET is usually defined by re-expression of E-cadherin, concomitant reduction in mesenchymal features, and re-differentiated metastatic foci. Of note, E-cadherin is itself required for the growth of primary tumors. Cancer cells with a terminal mesenchymal phenotype characterized by high ZEB1, ZEB2 and SNAI1, exhibited impaired tumor-initiating capacity. In contrast, CD49f^+^/EpCAM^+^/E-cadherin^hi^ cells had the highest tumor-initiating capacity in majority of tested TNBC PDXs, when compared to CD49f^+^/EpCAM^low^ and CD49f^low^/ EpCAM^low^ [165]. Among the first studies inspecting the mechanism of MET, Chao et al. demonstrated that E-cadherin-negative primary tumors originated from MDA-MB-231 cells, gave rise to E-cadherin positive metastatic foci in lungs. Moreover, modeling liver microenvironment by the co-culture of cancer cells with hepatocytes reactivated cancer cell epithelial phenotype and functional E-cadherin re-expression, suggesting the prominent role of the metastatic niche in MET activation [91]. These results have not yet been experimentally reproduced in vivo. Using a similar experimental model, MDA-MB-468, E-cadherin expression sustained in vitro and in vivo proliferation and was indispensable for formation of the lung and lymph node metastasis [166]. Conversely, loss of E-cadherin resulted in increased invasion capacity and dissemination, reduced cell survival and decreased colony formation capacity [167]. Another study linking MET and metastasis took advantage of a dynamic in vivo MDA-MB-468 model, employing Vimentin as an EMP marker. Compared to individually isolated cells or micrometastasis in lungs, lung macrometastasis exhibited more heterogeneous Vimentin and homogeneous E-cadherin expression as observed also in primary tumors. This suggests that MET likely occurred once overt metastases developed [168].

Independently, Dykxhoorn and colleagues demonstrated that only the epithelial, E-cadherin/miR-200/Cytokeratin-expressing subclone of 4T1 cells was capable to form macrometastatic liver and lung metastases, when compared to mesenchymal clone that effectively disseminated, but was only capable of forming micrometastatic lesions [145]. Similarly, re-expression of miR-200 followed by subsequent acquisition of epithelial traits and activation of the metastasis-suppressive program through SEC23A was critical for the formation of macrometastasis in lungs [144]. In the BT-549 model, reactivation of epithelial properties mediated by PRRX1 loss, upregulation of E-cadherin, and loss of Vimentin resulted in tumor-initiating ability and MET, followed by formation of the metastatic foci in lungs. The number of foci was increased in concomitant loss of PRRX1 and TWIST1. Activation of MET was further linked to the acquisition of stem-like properties, hence uncoupling stemness and EMT [24]. Similarly, epithelial features induced by the HLH TF inhibitor ID1, resulting in pulmonary metastasis, were associated with stem-like phenotype and acquired independently of EMT. Remarkably, ID1 was induced by TGF-β only in cells that previously underwent EMT, implying that EMT is a prerequisite for subsequent ID1-induced MET during lung colonization [169]. Tumor initiating abilities of plastic cells were also highlighted in a study by Kröger et al., who observed that cells residing in hybrid E/M state, as determined by SNAI1 and Wnt, formed tumors more effectively than cells in ‘locked’ E or M state. Interestingly, forced transition from hybrid E/M to fully mesenchymal state mediated by ectopic expression of ZEB1 was accompanied by the loss of tumorigenicity, implicating that the existence in the extremes of E/M spectrum is unfavorable for cancer cell aggressiveness [32]. As stressed in previous sections, reversion to the epithelial state is to a large extent orchestrated by microenvironmental tumor-stroma interactions in a context-dependent manner. This idea is supported by a study in a mouse model of TNBC, which demonstrated that the recruitment of bone marrow-derived myeloid progenitors to the pre-metastatic lungs was essential to induce a MET of DTCs and favor the subsequent formation of macrometastases [170].

## 4. Targeting Plasticity as a Therapeutic Strategy

Intratumoral heterogeneity and EMP as its prerequisite is one of the major challenges in targeting cancer. Both cells with activated EMT and CSC characteristics have been reported to confer drug resistance against multiple conventional therapeutics [29]. Moreover, mesenchymal, basal-like neoplasms tend to be more resistant to neoadjuvant chemotherapy than epithelial, luminal-like tumors [171,172]. The three main strategies have been proposed to combat plasticity: (i) targeting stimuli, which can prevent mesenchymal transitioning; (ii) targeting cells in mesenchymal or hybrid states and therefore prevent MET at secondary niche; (iii) reprogramming mesenchymal cells back to epithelial state [29]. For instance, in the first scenario, chemotherapy-treated TNBC breast cancer displayed enhanced TGF-β signaling and upregulated CSC-related genes. Mechanistically observed in vitro paclitaxel treatment increased IL-8 expression and expanded a CSC population where autocrine TGF-β signaling was active. Pharmacological inhibition using TGF-βR1 kinase inhibitor and clinical candidate galunisertib reduced the paclitaxel-resistant CSC population and prevented re-establishment of tumors after paclitaxel treatment in TNBC xenografts [173]. Moreover, inhibition of TGF-β signaling suppressed paclitaxel-induced EMT, and combined treatment employing TGF-β inhibitor EW-7197 improved the therapeutic effect of paclitaxel by decreasing the lung metastasis and increasing the survival in vivo [174]. Likewise, TGF-β inhibitors also proved their effectiveness in restricting docetaxel-induced EMT, and the combined treatment enhanced cytotoxic efficacy of doxorubicin [175]. The third strategy was demonstrated in study in which overexpression of miR-644a in cancer cells resulted in epithelial state of cells and increased response to doxorubicin mediated by miR-644a/CTBP1/p53 axis [176]. Pattabiraman et al. identified adenylate cyclase activators cholera toxin and forskolin through a compound screening as inducers of CDH1 transcription and MET activators [138]. Cells exposed to these substances reverted to an epithelial state, acquired junctional E-cadherin, became responsive to doxorubicin and paclitaxel treatment, and most importantly lost their tumor initiation capacity [138]. Re-expression of E-cadherin in TNBC has been also associated with decreased sensitivity to protein kinase inhibitor staurosporine and DNA topoisomerase I inhibitor camptothecin [90]. Treatment with eribulin, a non-taxane microtubule inhibitor, induced MET accompanied by decreased in vitro migration, as well as reduced metastatic potential in vivo [177]. Furthermore, a monoclonal antibody that neutralizes IL-8, HuMax-IL8, reverted mesenchymal phenotype in claudin-low TNBC models in vitro and in vivo, and decreased the recruitment of tumor-promoting polymorphonuclear myeloid-derived suppressor cells at the tumor site in xenografts. This effect was further enhanced by combination with docetaxel [178]. Finally, it has been reported that specific downregulation of PD-L1 in claudin-low breast cancer cells showed signs of EMT reversal, implying that patients might benefit from anti-PD-L1 targeted therapy [179]. One other emerging idea is to block plasticity by “fixing cells at a given position on the epithelial–mesenchymal axis” [180]; a potential way to “fix” the cells and block plasticity bi-directionally is to break positive feedback loops in underlying regulatory networks that allow state switching [181]. A proof-of-principle experimental validation of this idea was demonstrated by Celia-Terrassa et al. in a study where breaking the miR-200/ZEB feedback loop reduced metastasis in vivo [182], although further validation of this approach is needed.

Given these data, a combinatorial therapeutic approach leveraging from conventional drugs targeting proliferation and agents modulating cell plasticity might represent promising treatment strategies in the future. Some of the EMT-modulating drugs already tested in clinical trials are summarized in Table 1. However, optimal therapy timing, identification of potential biomarkers, and proper dissection of roles of particular states in tumor evolution are of utmost necessity for future clinical interventions [29].

## 5. Conclusions and Outlook

Recently accumulated data indicate that dynamic reciprocal transition between EMT and MET phenotypes represents the fundamental base of tumor progression in TNBC. Cancer cells comprising the triple-negative breast tumors are often enriched in mesenchymal traits when compared to other breast cancer subtypes, suggesting the presence of high degree of plasticity in TNBC. The EMT and MET programs, aberrantly activated by tumor cells and employed at different stages of cancer progression enable these cancer cells to dynamically change their phenotype, a feature that might be critical for successful metastatic dissemination and overcoming the multiple obstacles on route to a final destination-distant site parenchyma. EMT is considered an early event in breast cancer tumorigenesis, crucial for intravasation; MET, on the other hand, appears to be essential for subsequent re-activation in distant site and macrometastatic outgrowth. Multiple studies also underpinned the involvement of hybrid E/M states in aggressive behavior of tumor cells. However, some of the studies presented here relied solely on traditional molecular markers such as E-cadherin and Vimentin, whereas both EMT and MET programs display complex manifestation. Therefore, studies should identify these processes not only by well-known markers but also relate them to transcription factors and phenotypic features, such as proliferation and invasion. Moreover, enforced manipulations of gene expression usually used in plasticity studies artificially keep cells in fixed states and, therefore, do not recapitulate the whole dynamic EMT/MET spectrum of transient and reversible states. Conclusively, to precisely capture dynamics of the entire spectrum in time-dependent manner, development of more appropriate tools, such as inducible reporter systems or intra-vital imaging, and employment of multi-omics, single-cell based approaches together with integration of multi-scale computational modeling is required to fully reveal the extent of dynamics and implications of EMP in experimentally valid models and clinical specimens.

## Figures and Tables

**Table 1:** A list of drugs targeting EMT regulatory components in TNBC clinical trials.

Drug	Mechanisms	Clinical Trial ID	Monotherapy/Combination
Buparlisib	PI3K inhibitor	NCT02000882	Capecitabine
PF-03084014	Notch inhibitor	NCT02299635	Monotherapy
RO4929097		NCT01071564	Vismodegib
Fresolimumab	TGF-β blocking antibody	NCT01401062	Radiation therapy
Galunisertib	TGFβR1 kinase inhibitor	NCT02672475	Paclitaxel
Reparixin	IL-8 receptor CXCR1/2 inhibitor	NCT02370238	Paclitaxel
Entinostat	class I HDAC inhibitor of NF-κB, IL-6 and IL-8	NCT02708680	Atezolizumab
Everolimus	mTORC1 inhibitor reducing HIF-1α expression	NCT01931163	Cisplatin
Bicalutamide	androgen antagonist preventing AR-induced hypoxia	NCT03090165	Ribociclib
Cetuximab	EGFR inhibitor inhibiting synthesis of HIF-1α	NCT01097642	Ixabepilone
Lucitanib	angiogenesis inhibitor reducing MMPs and collagen	NCT02202746	Monotherapy
Vismodegib	Smoothened receptor antagonist, inhibiting Hedgehog signalling	NCT02694224	Paclitaxel Epirubicin Cyclophosphamide
Sonidegib		NCT01576666	Buparlisib
		NCT02027376	Docetaxel
ARRY-382	CSF1R inhibitor	NCT02880371	Pembrolizumab
Pexidartinib		NCT01596751	Eribulin
MEDI4736	anti-PD-L1 antibody	NCT02484404	Cediranib
		NCT02403271	Durvalumab
Vantictumab	Wnt pathway inhibitor	NCT01973309	Paclitaxel

## Data Availability

Not applicable.
